# Addressing Bias in Small RNA Library Preparation for Sequencing: A New Protocol Recovers MicroRNAs that Evade Capture by Current Methods

**DOI:** 10.3389/fgene.2015.00352

**Published:** 2015-12-22

**Authors:** Jeanette Baran-Gale, C. Lisa Kurtz, Michael R. Erdos, Christina Sison, Alice Young, Emily E. Fannin, Peter S. Chines, Praveen Sethupathy

**Affiliations:** ^1^Bioinformatics and Computational Biology Curriculum, School of Medicine, University of North Carolina at Chapel HillChapel Hill, NC, USA; ^2^Department of Genetics, School of Medicine, University of North Carolina at Chapel HillChapel Hill, NC, USA; ^3^National Human Genome Research Institute, National Institutes of HealthBethesda, MD, USA; ^4^NIH Intramural Sequencing Center, National Institutes of HealthRockville, MD, USA

**Keywords:** small RNA library preparation, adapter ligation bias, microRNA, sequencing, adapter dimers

## Abstract

Recent advances in sequencing technology have helped unveil the unexpected complexity and diversity of small RNAs. A critical step in small RNA library preparation for sequencing is the ligation of adapter sequences to both the 5′ and 3′ ends of small RNAs. Studies have shown that adapter ligation introduces a significant but widely unappreciated bias in the results of high-throughput small RNA sequencing. We show that due to this bias the two widely used Illumina library preparation protocols produce strikingly different microRNA (miRNA) expression profiles in the same batch of cells. There are 102 highly expressed miRNAs that are >5-fold differentially detected and some miRNAs, such as miR-24-3p, are over 30-fold differentially detected. While some level of bias in library preparation is not surprising, the apparent massive differential bias between these two widely used adapter sets is not well appreciated. In an attempt to mitigate this bias, the new Bioo Scientific NEXTflex V2 protocol utilizes a pool of adapters with random nucleotides at the ligation boundary. We show that this protocol is able to detect robustly several miRNAs that evade capture by the Illumina-based methods. While these analyses do not indicate a definitive gold standard for small RNA library preparation, the results of the NEXTflex protocol do correlate best with RT-qPCR. As increasingly more laboratories seek to study small RNAs, researchers should be aware of the extent to which the results may differ with different protocols, and should make an informed decision about the protocol that best fits their study.

## Introduction

Small RNAs, such as microRNAs (miRNAs), are important regulators of gene expression in a wide variety of normal biological and pathological processes (Couzin, 2008; Bartel, 2009). Numerous technologies, including quantitative PCR (qPCR), microarray, and deep sequencing, are presently in use for high-throughput miRNA profiling (Baker, 2010; Gunaratne et al., 2012; Pritchard et al., 2012). Though each of these methods has both advantages and limitations, deep sequencing has emerged as the gold standard for discovery and quantification of miRNAs, particularly for those that are of low abundance. Numerous small RNA library preparation protocols are currently available, including kits from Illumina, Applied Biosystems (ABI) SOLiD, New England BioLabs (NEB), and TriLink Biotechnologies (**Table 1**). Recent studies have demonstrated that each of these technologies harbors different limitations that lead to variable biases (Linsen et al., 2009; Tian et al., 2010).

**Table 1:** Current small RNA library preparation protocols and features.

Company	Protocol	Adapters	Adapter dimer removal	RNA input recommendations
Illumina	**V1.5**	Fixed	None	1-10 μg total RNA
Illumina	**TruSeq**	Fixed	None	1 μg total RNA
Applied BioSystems	SOLiD small RNA expression kit	Degenerate hybridization	None	0.25-1 μg total RNA
Bioo Scientific	**NEXTflex V2**	Degenerate	Excess 3′ adapter removal	1-10 μg total RNA
NEB	NEBNext	Fixed	Excess 3′ adapter removal	0.1-1 μg total RNA
TriLink Biotechnologies	CleanTag	Fixed	Chemically modified adapters	1 ng-1 μg total RNA
SeqMatic	TailorMix miRNA V2	Fixed	Advanced gel extraction	> 10 ng total RNA

*The protocols discussed in this study are in boldface font.*

A critical step in the preparation of a small RNA library for deep sequencing is the ligation of adapter sequences to both ends of small RNAs. These adapters provide the template for primer-based reverse transcription, amplification, and sequencing. The efficiency of adapter ligation to small RNAs is thought to depend on the adapter sequence, the ligase, and the nucleotide composition and secondary structures of the small RNAs (Hafner et al., 2011; Jayaprakash et al., 2011; Zhuang et al., 2012; Fuchs et al., 2015). Differences in adapter ligation efficiency among available protocols can drastically alter the perceived abundance of individual miRNAs.

Currently, the most widely used library preparation kits are those provided by Illumina. Illumina introduced the v1.5 small RNA library preparation method in February 2009 and the TruSeq method in November 2010. Because one critical difference between these methods is the adapter sequences, some level of differential bias between these two methods is expected. However, the extent of the bias has not been evaluated previously and could be important for guiding accurate comparison of miRNA expression results between these two methods.

**FIGURE 1:**
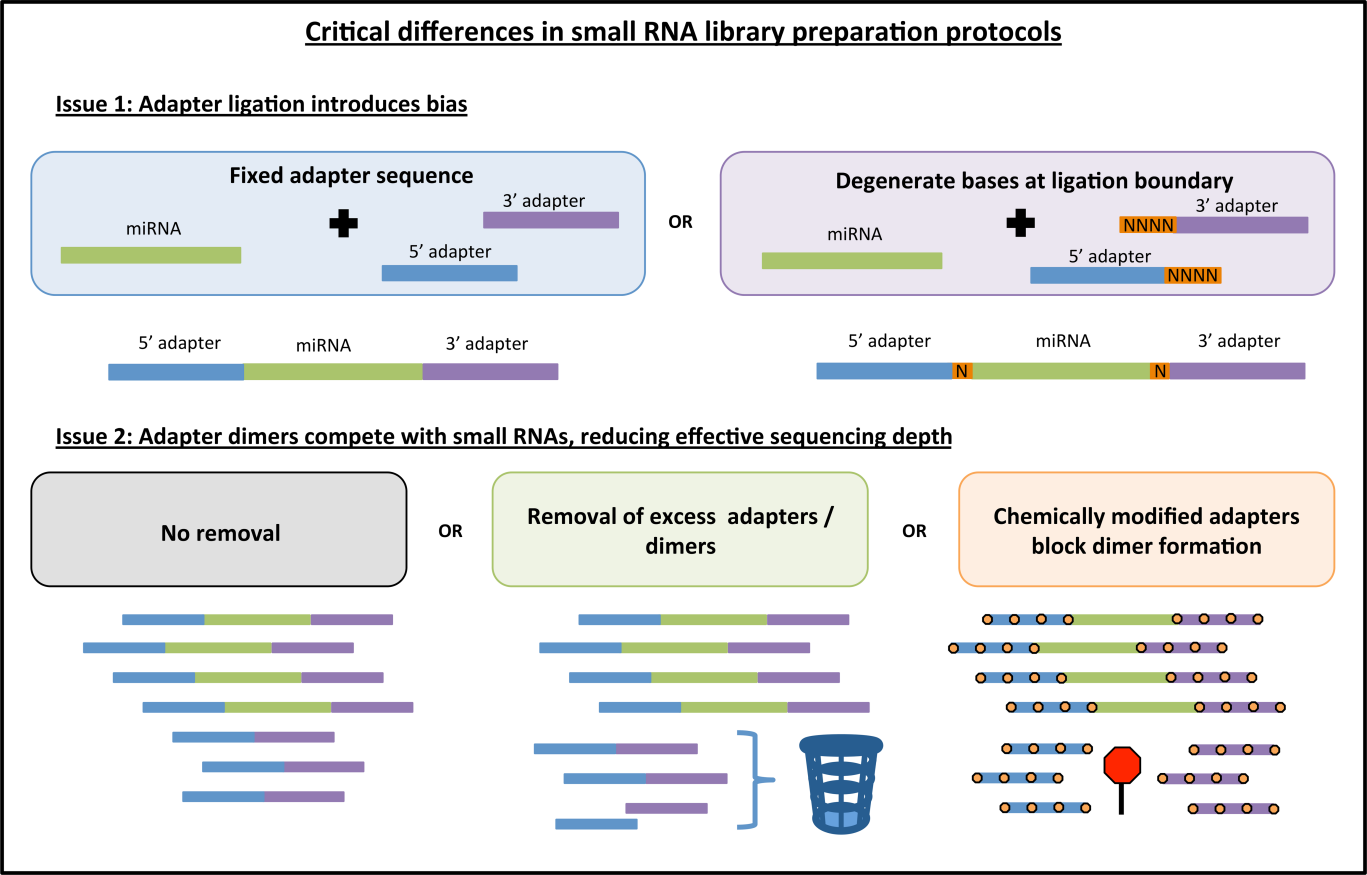
**Key differences among different commercially available library preparation kits for small RNA sequencing.** Some of the innovations in small RNA library preparation are highlighted here. First, current kits either use fixed adapter sequences or they introduce degenerate bases to both the 3′ and 5′ ligation boundary to improve adapter ligation efficiency. Second, adapter dimers can be generated causing a portion of sequenced reads to contain no insert. These dimers can be blocked or removed, thus increasing effective sequencing depth. Note: orange boxes indicating degenerate bases are not depicted in the adapter dimer graphic for the sake of simplicity.

To address this bias, a few new library preparation methods have been proposed. For example, one new protocol called NEXTflex V2 from Bioo Scientific uses a pool of adapters, each with random nucleotides (degenerate bases) at the ligation boundary (**Figure 1**). The idea behind this strategy is to increase the diversity of adapter sequences thereby increasing the chance that any given miRNA will be able to ligate efficiently, and thus mitigating the overall bias inherent to protocols that use only one set of adapters. As another example, a recent study (Fuchs et al., 2015) uses a 5′ adapter with a short subsequence that is fully complementary to a region within the 3′ adapter. The intent of this method is to encourage all ligated miRNAs to form the same circular RNA structure and thus mitigate structure-based bias across miRNAs.

Another important issue for small RNA library preparation is the formation of adapter dimers (**Figure 1**). An abundance of adapter dimers in small RNA libraries can lead to sequencing a substantial number of reads with no miRNA insert, thus effectively reducing the proportion of informative sequencing reads (Kawano et al., 2010). Currently available library preparation kits either (1) fail to address this issue or suggest more precise gel cutting to avoid the adapter dimer band, (2) use some method of eliminating excess 3′ adapter prior to the 5′ adapter ligation step, or (3) use chemically modified adapters that inhibit the formation of adapter dimers (**Figure 1**). Kits that address the issue of adapter dimers can typically produce high-quality results with a lower abundance of input RNA. As the field moves toward sequencing of sub-populations of cells or single cells, the adapter dimer issue will become increasingly important.

In this study, we directly compare the small RNA sequencing results between Illumina v1.5 and TruSeq. We also perform the sequencing on two different Illumina platforms (GAIIx and HiSeq) and at two different sequencing centers (UNC and NIH). While we expected some level of bias in the library preparation, the apparent extensive differential bias between these two widely used Illumina adapter sets is striking and not reported previously. For example, 50 highly expressed miRNA species are >10-fold differentially detected between v1.5 and TruSeq. This finding serves as an important caution, particularly to laboratories/facilities that used v1.5 but are now transitioning to the newer protocol. Finally, we compare these results to a library generated by a new protocol from Bioo Scientific that seeks to combat both adapter ligation bias and excessive adapter dimer formation. We show that this new protocol is able to detect miRNAs that evade capture by the more commonly used Illumina protocols, and also produces miRNA expression counts that are highly correlated with measurements acquired by RT-qPCR. The findings of this study add to the growing body of literature on bias in small RNA sequencing that merits continued investigation, particularly with regard to the development of strategies for bias remediation and improved miRNA quantification.

## Materials and Methods

### Sequencing and Bioinformatic Analysis

Mouse insulinoma (MIN6) cells were cultured as previously described (Baran-Gale et al., 2013). Cells were lysed and RNA was isolated using either the Norgen (ON, Canada) Total RNA Purification Kit (UNC) or TRIzol-mediated extraction (NIH). Only samples with an RNA Integrity Number (RIN) of 8 or higher, as measured by Agilent (Santa Clara, CA, USA) Bioanalyzer 2100, were considered for further analysis. Small RNA libraries were generated using either the Illumina v1.5 protocol or the Illumina TruSeq protocol. Single-end sequencing was performed on either the Illumina GAIIx or Illumina HiSeq 2000 platforms. One library was also generated using the Bioo Scientific NEXTflex V2 protocol. For libraries generated with either of the Illumina protocols, small RNA-seq reads were trimmed using cutAdapt (-O 10 –e 0.1) to remove remnants of the 3′-adapter sequence. For the library generated with the NEXTflex protocol, the first four and last four nucleotides of small RNA-seq reads were trimmed to remove the degenerate nucleotides in the adapters. Subsequent mapping of trimmed reads to the mouse genome and miRNA/isomiR quantification were performed exactly as previously described (Baran-Gale et al., 2013).

A 9 week old C57BL/6J female mouse was purchased from Jackson Laboratories (Bar Harbor, ME, USA) as part of a cohort of control mice for another study. This mouse was maintained on a 12 h light/dark cycle with access to a standard chow diet and H_2_O *ad libitum.* After a 10 day acclimation period, the control animal was weighed and injected via tail vein with RNase-free sterile saline (Bioo Scientific; Austin, TX, USA). Seven days after dosing with saline, the animal was fasted (overnight), sacrificed by cervical dislocation without anesthesia and organs were collected. The liver was flash frozen in liquid nitrogen and stored at -80°C until RNA was extracted using the Norgen (ON, Canada) Total RNA Purification kit. Libraries were generated using either the Illumina TruSeq or Bioo Scientific NEXTflex V2 protocols. All animal work was performed in accordance with the Public Health Service Policy on Humane Care and Use of Laboratory Animals, and all studies were approved by the Institutional Animal Care and Use Committee (IACUC) at the University of North Carolina at Chapel Hill.

### Real Time Quantitative PCR Analysis and Linear Regression

Mouse insulinoma cells were cultured and lysed as above and RNA was isolated using the Norgen Total RNA Purification Kit. Complementary DNA (cDNA) was synthesized using the TaqMan miRNA Reverse Transcription kit (Applied Biosystems; Grand Island, NY, USA) according to the manufacturer’s instructions. Real-time PCR amplification was performed using TaqMan miRNA assays in TaqMan Universal PCR Master Mix on a BioRad CFX96 Touch Real Time PCR Detection system (Bio-Rad Laboratories, Inc., Richmond, CA). Reactions were performed in triplicate using *U6* as the internal control. miRNA levels were expressed as relative quantitative values, which represent fold differences relative to miR-30e-5p (a miRNA we found to be among the least variable in expression across most library preparation protocols). All TaqMan assays used in this study where purchased from Applied Biosystems, Inc. (Grand Island, NY, USA) and include: mmu-miR-24-3p (4427975-000402), mmu-miR-27b-3p (4427975-000409), mmu-miR-29a-3p (4427975-002112), mmu-miR-375-3p (4427975-000564), miR-30e-5p (4427975-002223), and *U6* (4427975-001973).

Linear regression was used to examine the relationship among different miRNA detection methods (RT-qPCR, Illumina V1.5, Illumina TruSeq and Bioo Scientific NEXTflex V2) in terms of the expression levels of five miRNAs (miR-129-5p, miR-24-3p, miR-27b-3p, miR-29a-3p, and miR-375-3p). For this analysis the expression level of each miRNA was normalized to that of miR-30e-5p (a miRNA we found to be among the least variable in expression across most library methods). A linear model was created in which the relative expression as measured by method Y (RE_Y_) was modeled as a function of the relative expression as measured by method X (RE_X_). In this model (RE_Y_ = α + β ^∗^ RE_X_ + 𝜀), the term α represents the estimated expression level using method Y when the expression level is 0 using method X, β represents the weight applied to the expression as measured by method X, and 𝜀 represents the random error in the model. To assess the model fit, two additional factors are computed and shown: *R*^2^ (the fraction of variance that is explained by the model) and σ^2^ (the estimated variance of the random error, 𝜀).

## Results

We isolated RNA from a widely used pancreatic beta-cell-like cell line (MIN6) and performed small RNA-seq using four different methods: (1) Illumina v1.5 library preparation sequenced on GAIIx platform (v1.5-GAIIx), (2) Illumina TruSeq library preparation sequenced on GAIIx platform (TS-GAIIx), (3) Illumina TruSeq library preparation sequenced on HiSeq platform (TS-HiSeq), and (4) Bioo Scientific NEXTflex V2 library preparation sequenced on the HiSeq platform (NF-HiSeq). TS-GAIIx and v1.5GAIIx were carried out at the NIH Intramural Sequencing Center (NISC) on June 25^th^, 2013; TS-HiSeq was performed at the UNC High throughout Sequencing Facility (HTSF) on June 6th, 2013; and NF-HiSeq was performed at the Genome Sequencing Facility (GSF) at Greehey Children’s Cancer Research Institute (GCCRI) in University of Texas Health Science Center at San Antonio (UTHSCSA) on March 24th, 2015. Three replicate small RNA libraries were generated for each of the first three methods, and one replicate was generated for the fourth method, yielding a total of ten small RNA-seq datasets (Supplementary Table S1). The NEXTflex library was prepared from the same RNA that was used to prepare one of the TruSeq libraries.

We used our previously published bioinformatic pipeline (Baran-Gale et al., 2013) to analyze the small RNA-seq reads in each dataset. Results of the 3′-adapter trimming and genome mapping are provided in Supplementary Table S1. The total number of reads across the ten datasets range from ∼17 million to ∼29 million (Supplementary Table S1). In each of the datasets, >70% of the alignable reads map to annotated miRNAs and >1000 distinct mature miRNAs are represented by at least ten reads. Among these miRNAs, 358 have a relative expression of at least 100 reads per million mapped reads (RPMM) in at least one library (Supplementary Table S2). We refer to these miRNAs as “highly expressed.” To compare miRNA expression profiles across datasets, we correlated the expression profiles of these abundant miRNAs across all ten datasets.

**FIGURE 2:**
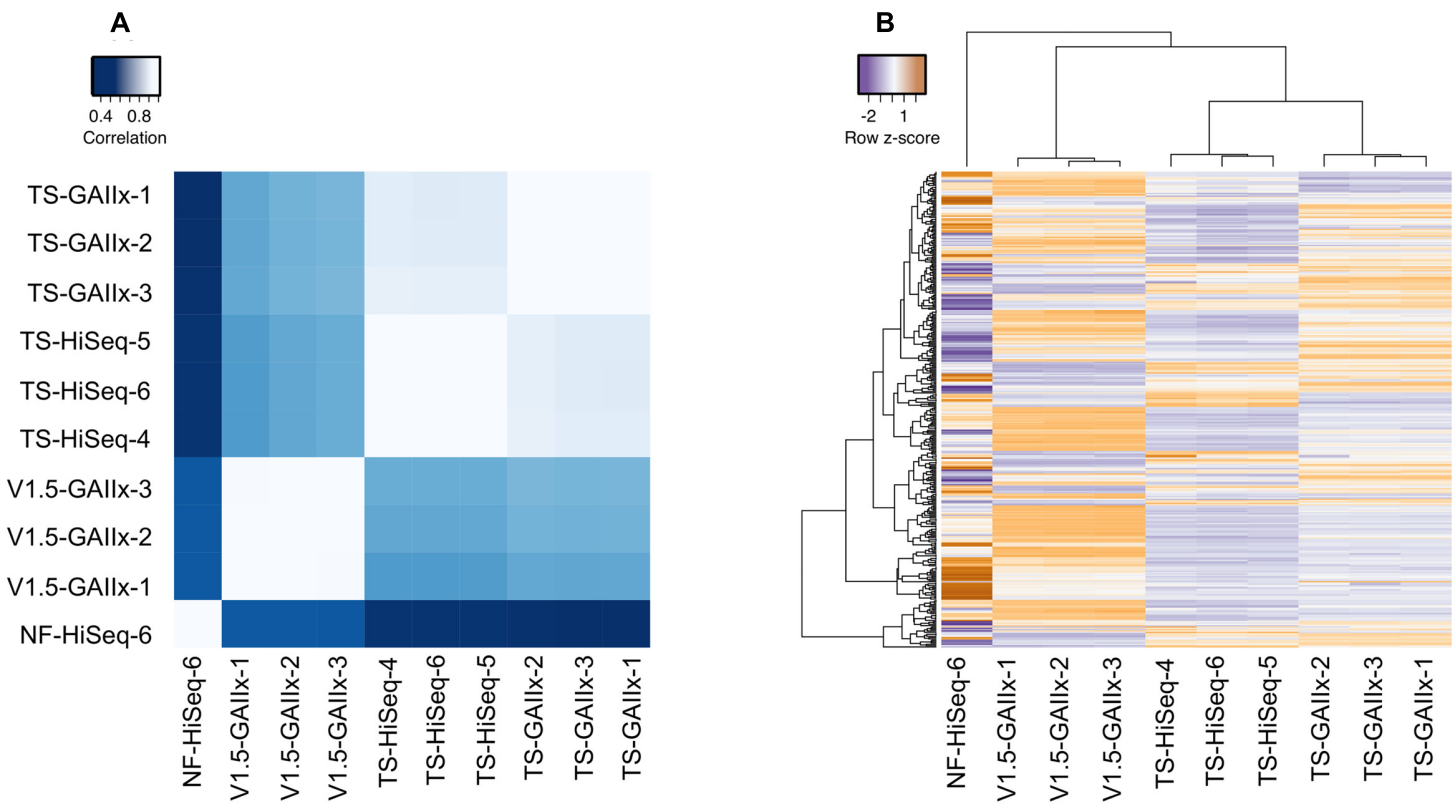
**Comparison of miRNA expression profiles among different library preparation protocols reveals massive differential bias.** A comparison of the following four methods is shown: Illumina v1.5 library preparation sequenced on GAIIx platform (v1.5_GAIIx), Illumina TruSeq library preparation sequenced on GAIIx platform (TS_GAIIx), Illumina TruSeq library preparation sequenced on HiSeq platform (TS_HiSeq) and Bioo Scientific NEXTflexV2 library preparation sequenced on the HiSeq platform (NF-HiSeq). Three biological replicate small RNA libraries were generated for each of the first three methods and one replicate was generated for the NF-HiSeq method. **(A)** Correlation of miRNA profiles between each pair of datasets (correlation values were calculated by Pearson’s metric). Similar results were obtained with Spearman’s correlation coefficient, rho (data not shown). White and blue colors indicate strongest and weakest correlation, respectively. **(B)** miRNA expression profiles across all 10 samples. Hierarchical clustering was used to identify samples with closely related expression profiles. Expression is represented as *z*-score, indicating the number of standard deviations below (purple) or above (orange) the mean across all ten libraries. Both **(A,B)** used only the set of miRNAs identified as “highly expressed” (*n* = 358).

The miRNA expression profiles from biological replicates within each method are very highly correlated (average pairwise *r*^2^ > 0.99), clearly demonstrating that both the method of library preparation and the sequencing platform yield exceptionally reproducible results (**Figure 2A**). Furthermore, we also observe a very strong correlation (average pairwise *r*^2^ > 0.86) among TS-GAIIx and TS-HiSeq samples, but substantially lower correlation (average pairwise *r*^2^ ∼ 0.43) among TS-GAIIx (or TS-HiSeq) and v1.5-GAIIx samples (**Figure 2A**). These results indicate that neither sequencing platform (GAIIx vs. HiSeq) nor sequencing facility (UNC vs. NIH) is a major contributor to technical variation, but that the method of library preparation (TS vs. v1.5) is a significant factor (**Figure 2B**).

**FIGURE 3:**
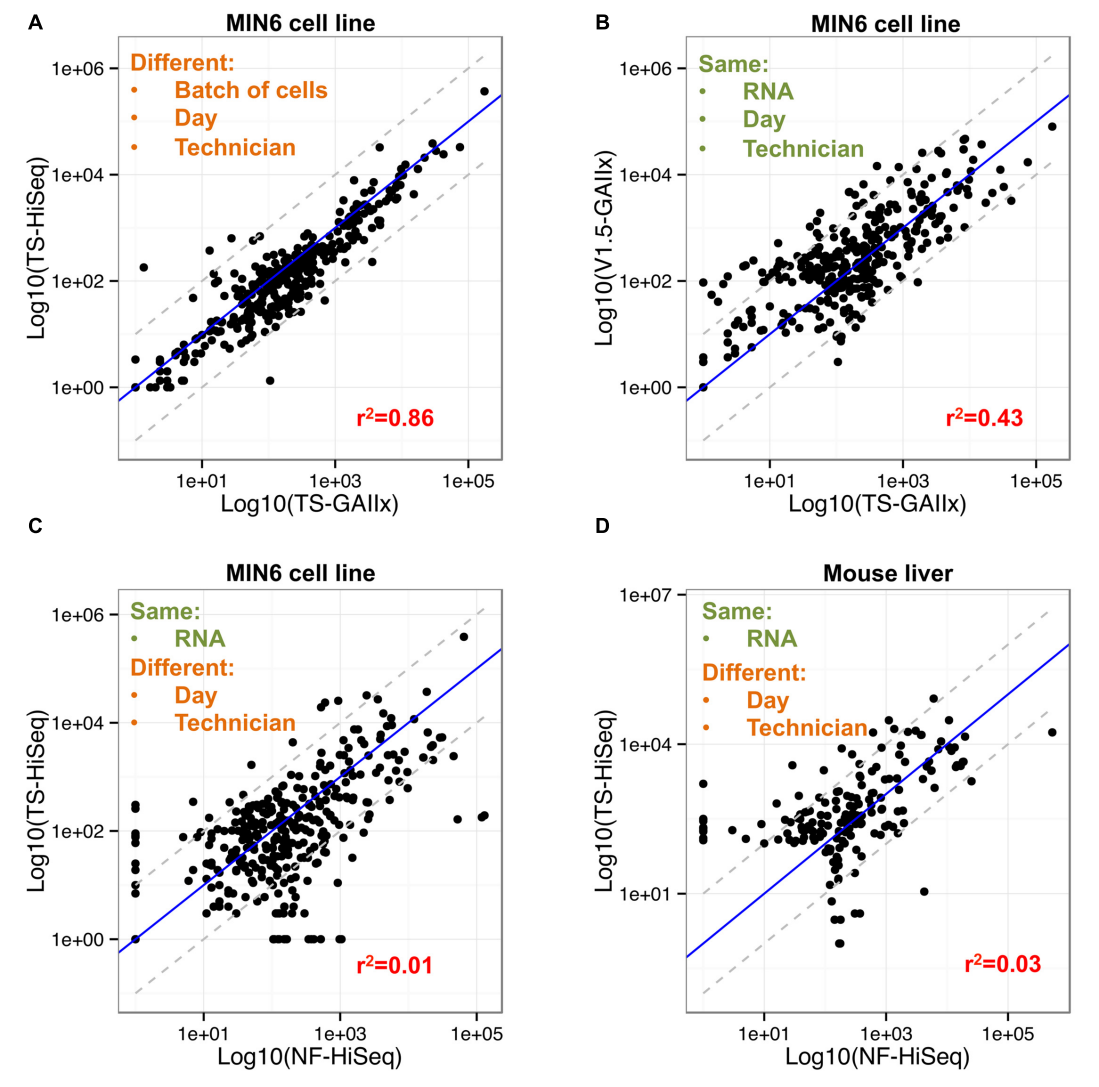
**Fifty of the most abundant miRNAs are greater than ten-fold differentially detected between Illumina v1.5 and TruSeq. (A)** Comparison of relative expression levels of miRNAs in MIN6 (*n* = 358) between the GAIIx and HiSeq sequencing platforms with libraries prepared by TruSeq (TS) is shown. Each data point represents the average relative expression level for an individual miRNA across three biological replicates. **(B)** Comparison of relative expression levels of miRNAs in MIN6 (*n* = 358) between the v1.5 and TruSeq (TS) library preparation methods is shown. Each data point represents the average relative expression level for an individual miRNA across three biological replicates. **(C,D)** Comparison of relative expression levels of miRNAs in MIN6 (*n* = 358, **C**) and mouse liver (*n* = 178, **D**) between the TruSeq (TS) and NEXTflex (NF) library preparation methods is shown. Each data point represents the average relative expression level for an individual miRNA across three biological replicates **(A,B)**, or one biological replicate **(C,D)**. Relative miRNA expression levels were calculated according to the following: log_10_ (mean(miRNA RPMM)), where RPMM is reads per million mapped reads. Pearson correlation values are displayed in red text within each panel, and gray dashed lines denote 10-fold differential expression.

Only 7 out of the 358 highly expressed miRNAs included in the correlation analysis are >10-fold differentially detected between TS-GAIIx and TS-HiSeq (**Figure 3A**, Supplementary Figure S1). Moreover, most of these differentially detected miRNAs are on the lower end of the expression spectrum (Supplementary Table S2). In stark contrast, when comparing TS-GAIIx with v1.5-GAIIx, 50 miRNAs are >10-fold differentially detected and 102 are >5-fold differentially detected (**Figure 3B**). Strikingly, ∼80% (*n* = 40/50) of the former and ∼74% (*n* = 75/102) of the latter set of miRNAs are present at greater abundance in the samples prepared by v1.5 compared to the samples prepared by TruSeq (**Figure 3B**). These miRNAs include several that are known regulators of beta cell development and function, including miR-24-3p (Zhu et al., 2013), miR-29b-3p (Pullen et al., 2011), and miR-200c-3p (Liao et al., 2013), which are ∼36-fold, ∼31-fold, and ∼13-fold more highly detected in the samples prepared by v1.5, respectively. miR-24-3p is among the ten most highly expressed miRNAs in MIN6 cells according to v1.5, but is consistently not even in the top hundred according to TruSeq (Supplementary Table S2). It is worth noting that despite the overall bias toward higher miRNA expression levels in samples prepared by v1.5, a few miRNAs are more highly detected in samples prepared by TruSeq (**Figure 3B**). For example, miR-26a-5p, which is known to have functional relevance in the beta cell (Melkman-Zehavi et al., 2011), is among the ten most highly expressed miRNAs in MIN6 cells according to TruSeq, but is scarcely in the top fifty according to v1.5 (Supplementary Table S2).

We also used the new Bioo Scientific NEXTflex V2 protocol to prepare and sequence another small RNA library (NF-HiSeq-6) from the same MIN6 RNA that we had used previously for the preparation of a library by TruSeq (TS-HiSeq-6). The miRNA expression profiles produced by the two different library preparation methods are very poorly correlated (*r*^2^ ∼ 0.1; **Figure 2A**). The miRNA profile produced by NEXTflex V2 is completely different from that of Illumina v1.5 as well (**Figures 2A,B**). A total of 75 out of the 358 highly expressed miRNAs, including several with important functions in pancreatic beta cells, are > 10-fold differentially detected between TS-HiSeq and NF-HiSeq (**Figure 3C**). For example, the miR-7 family of miRNAs, which regulates insulin secretion in beta cells (Latreille et al., 2014), evades detection by the Illumina library preparation methods but is robustly detected by the NEXTflex V2 protocol. Strikingly, miR-7a-3p is ∼670-fold more highly detected by NEXTflex V2 than by TruSeq, and ∼50-fold more highly detected by NEXTflex V2 than by v1.5. Other miRNAs implicated in the control of beta cell function such as let-7b-5p (Frost and Olson, 2011; Pullen et al., 2011; Roggli et al., 2012) and miR-24-3p (Zhu et al., 2013) are ∼19-fold and ∼15-fold more highly detected by NEXTflex V2 than by TruSeq, respectively. It is worth noting that not all miRNAs are more highly detected by the NEXTflex V2 method. For example, miR-375-3p (another miRNA critical to beta cell function; Poy et al., 2004, 2009) is detected at levels ∼6-fold lower by NEXTflex V2 than by TruSeq, although it is still identified as one of the most highly expressed miRNAs.

To test whether the differences in miRNA expression profiles between TruSeq and NEXTflex V2 library preparation methods are unique to MIN6 or cell culture, we repeated the analysis with RNA from mouse liver tissue. Specifically, we prepared two separate small RNA libraries, using the TruSeq and NEXTflex V2 protocols, from the same mouse liver RNA and then performed sequencing on the HiSeq platform. Out of the 178 highly expressed miRNAs in the mouse liver, 40 were >10-fold differentially detected between the TS-HiSeq-ML and NF-HiSeq-ML libraries (**Figure 3D**; Supplementary Table S3). Included in this list is miR-122, which has a critical role in liver biology and disease (Tsai et al., 2012; Bandiera et al., 2015). This miRNA is detected ∼31-fold more highly with NEXTflex V2 protocol. In sum, ∼20% of highly detected miRNAs are >10-fold differentially detected between the two protocols in both cell lines (MIN6) and primary tissue (mouse liver).

**FIGURE 4:**
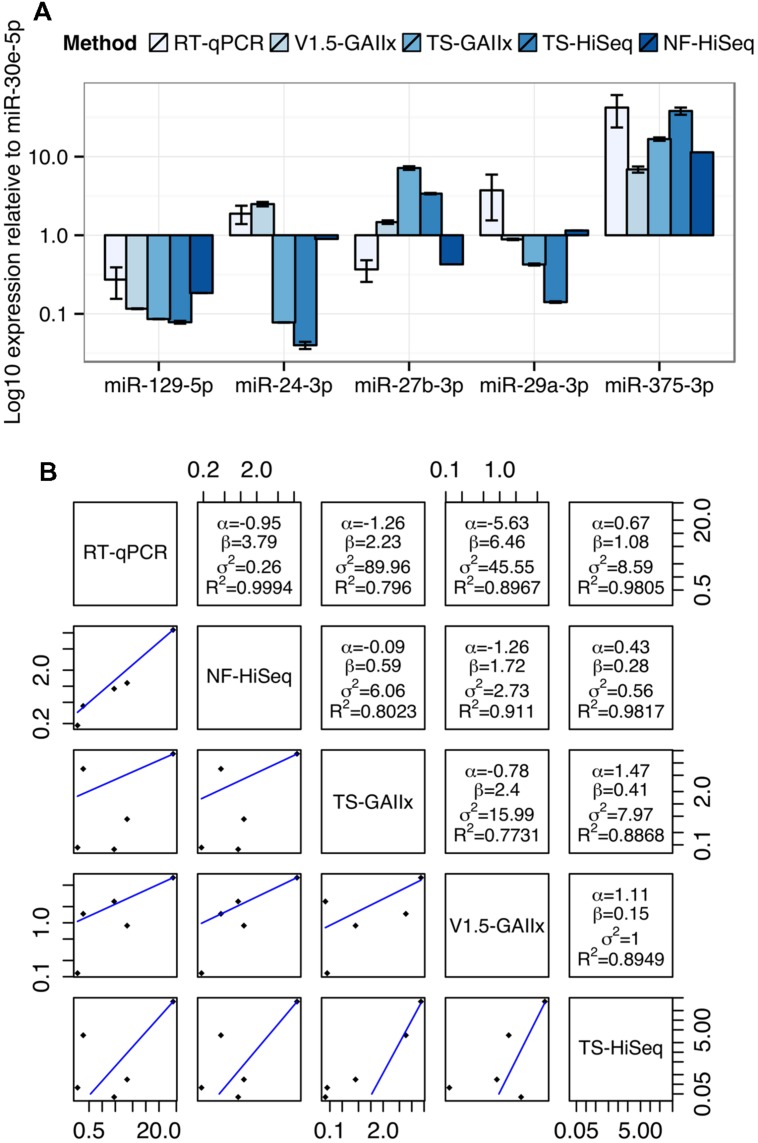
**Measurements by quantitative PCR are best correlated with NEXTflex V2. (A)** Comparison of relative expression levels of four miRNAs (miR-24-3p, miR-27b-3p, miR-29a-3p, and miR-375-3p) across five different methods of miRNA detection is shown. **(B)** Regression analysis of the relative expression of four miRNAs for each pair of detection methods is shown. The linear regression line is shown below the diagonal and the linear model parameters are shown above the diagonal. miRNA expression levels were normalized to miR-30e-5p, which represents a housekeeping miRNA due to its invariance and robust expression across most datasets. Linear model parameters: α = intercept, β = coefficient, σ^2^ = squared residual error, *R*^2^ = fraction of variance explained by model.

Finally, we selected five miRNAs highly expressed in MIN6 cells, miR-129-5p, miR-24-3p, miR-27b-3p, miR-29a-3p, and miR-375-3p for quantification by TaqMan-based real time reverse transcriptase quantitative PCR (RT-qPCR; **Figure 4A**). To facilitate a comparison of the findings between RT-qPCR and the sequencing methods, we normalized the expression levels of each miRNA to that of miR-30e-5p, which is highly expressed and among the least variable across the ten small RNA-seq datasets. The sequencing method that the RT-qPCR results more closely resemble depends on the miRNA. For example, qPCR-based expression for miR-24-3p best matches that of v1.5, whereas for miR-27b-3p it best matches that of NEXTflex V2. We next generated a mathematical model to describe the linear relationship in miRNA expression between each pair of miRNA detection methods (**Figure 4B**). The residual error values (σ^2^) are by far the lowest for the model relating RT-qPCR and NEXTflex V2, indicating that these two methods are the best correlated. As RT-qPCR experiments are not without their own biases, it is important to note that these data do not prove definitively that NEXTflex V2 is the most accurate library preparation protocol. However, the data do suggest that the NEXTflex V2 protocol is indeed mitigating the adapter ligation bias inherent to the other protocols.

## Discussion

The presence of bias in small RNA profiling is well established in the literature (Alon et al., 2011; Hafner et al., 2011; Van Nieuwerburgh et al., 2011; Linsen and Cuppen, 2012). Differential bias across various expression platforms (e.g., microarray, qPCR, sequencing) and sequencing technologies (e.g., Illumina, ABI SOLiD, 454 Life Sciences) has also been demonstrated (Linsen et al., 2009; Willenbrock et al., 2009; Tian et al., 2010). However, no study has focused on different library preparation methods within the same sequencing technology. Here we compare two of the most popular methods from Illumina (v1.5 and TruSeq). The results of our study point to a massive differential miRNA detection bias between these two library preparation methods. This finding was independent of the sequencing center (NIH, UNC) and sequencing platform (GAIIx, HiSeq). While some level of bias in library preparation is not surprising, the apparent extensive differential bias between these two widely used adapter sets is striking and not well appreciated (for example, miR-24-3p was detected very highly in the v1.5 libraries but was almost nonexistent in the TruSeq libraries).

Although we believe the extent of the bias remains poorly appreciated among many small RNA researchers, this bias has been investigated in a few studies, which together conclude that ligation efficiency is strongly affected by the co-fold structure of the target RNA and the adapter. Van Nieuwerburgh et al. (2011) demonstrated that sequencing of identical samples prepared with different barcodes at the 5′ ligation boundary led to poor reproducibility, in contrast to methods in which the barcode is embedded within the adapter itself (such as TruSeq). This finding suggests that sequence diversity at the ligation boundaries could lead to variable efficiency of adapter ligation, which in turn would result in significant but artefactual effects on miRNA detection and quantification. A subsequent study by Jayaprakash et al. provided further support for this finding, as they showed that certain miRNA species could be captured effectively only using a scheme that by introducing random bases at the ligation boundary. Specifically, this study concluded that introducing two random bases at both the 5′ and 3′ ligation boundaries could capture most miRNA species, but that at least one miRNA (miR-106b) required four random bases at the 5′ ligation boundary in order to be captured efficiently. Other studies (Hafner et al., 2011; Sorefan et al., 2012; Zhuang et al., 2012; Fuchs et al., 2015) have investigated the contribution of RNA structure to the adapter ligation bias issue. Sorefan et al. (2012) found that the introduction of four degenerate bases to both the 3′ and 5′ ligation boundaries increased the diversity of structures produced by the adapter and target sequence, and thereby reduced adapter ligation bias. Zhuang et al. (2012) showed that certain RNA/adapter co-fold structures are preferred by a variety of T4 RNA ligases, but observed no sequence bias. Together these studies suggest that introducing degenerate bases to both ligation boundaries introduces both sequence and structural diversity that improves adapter ligation likely by introducing favored RNA/adapter co-fold structures.

Very recently, several new commercially available small RNA library preparation protocols have been introduced. Of these new methods, only the Bioo Scientific NEXTflex V2 protocol also addresses the important issue of adapter dimer formation. In our studies, we found that NEXTflex V2 is able to detect robustly several functionally important miRNAs that partially or completely evade detection by the widely used Illumina library preparation protocols. A prominent example of this in MIN6 cells is miR-7a-3p, which plays a critical role in beta cell function. Moreover, we show that miRNA expression levels according to NEXTflex V2 are very highly correlated with RT-qPCR measurements. While we cannot say that the results of the NEXTflex V2 method accurately represents the “absolute” expression levels of miRNAs, the results of our analysis lead us to suggest that this protocol provides the least biased measure of miRNA expression among the tested methods.

It is important to note that our study does not suggest that one method of library preparation is necessarily always more reliable or accurate for miRNA detection than the other. Because the ligation efficiencies of different adapter sequences may differ based on features that vary across miRNAs, such as nucleotide sequence, chemical modification, and secondary structure (Linsen et al., 2009; Hafner et al., 2011; Sorefan et al., 2012), care must be taken when using methods that utilize fixed adapter sequences. As the factors that control the differential biases between adapter sets continue to be investigated, we expect to see continued innovation in small RNA library preparation protocols. Researchers seeking to ameliorate the influence of adapter ligation biases on miRNA expression levels can consider using protocols that utilize degenerate bases at the adapter ligation boundaries (**Table 1**). No one protocol fits every experiment; for example, experiments with limited input RNA are better off selecting protocols optimized for such samples regardless of adapter bias considerations.

As increasingly more laboratories begin sequencing small RNAs, researchers should be aware of the extent to which the results may differ from previously published results (depending on the protocol used). We strongly caution researchers against merging together small RNA-seq data generated from different adapter sequences. Also, in any standard small RNA-seq study in which only one adapter set is used for library preparation, one should be aware of the potential pitfalls of applying arbitrary cutoffs based on expression (such as “top 100 detected”) to identify miRNAs for further functional analysis, because some miRNAs that appear lowly expressed could be inefficiently detected for purely technical reasons (such as miR-24-3p in the TruSeq datasets presented in this study). In general, we recommend against using small RNA-seq data to make calls on the “absolute” levels of miRNAs, unless additional precaution has been taken to substantially mitigate the biases discussed here. Despite these issues, deep sequencing is still an extremely valuable method for *de novo* discovery of isomiRs and novel small RNAs, as well as for studying relative miRNA expression changes across different conditions or time points.

## Data Availability

MIN6 TS_HiSeq reads are available at Gene Expression Omnibus (GEO) under the accession number GSE44262. MIN6 v1.5_GAIIx, TS_GAIIx, NF_HiSeq, and both mouse liver libraries will also be made available at GEO (GSE75457).

## Author Contributions

This study was conceived of by JG and PS in collaboration with ME and PC. JG performed all UNC based experiments with the aid of CLK or EEF, and analyzed all data. CS and AY were responsible for sequencing performed at the NIH. JG and PS wrote the manuscript.

## Conflict of Interest Statement

The authors declare that the research was conducted in the absence of any commercial or financial relationships that could be construed as a potential conflict of interest.
