# Supplementary figures and images for: Addressing Bias in Small RNA Library Preparation for Sequencing: A New Protocol Recovers MicroRNAs that Evade Capture by Current Methods
Source: Front Genet. 2015 Dec 22;6:352. doi: 10.3389/fgene.2015.00352 (PMC4686641; doi:10.3389/fgene.2015.00352)

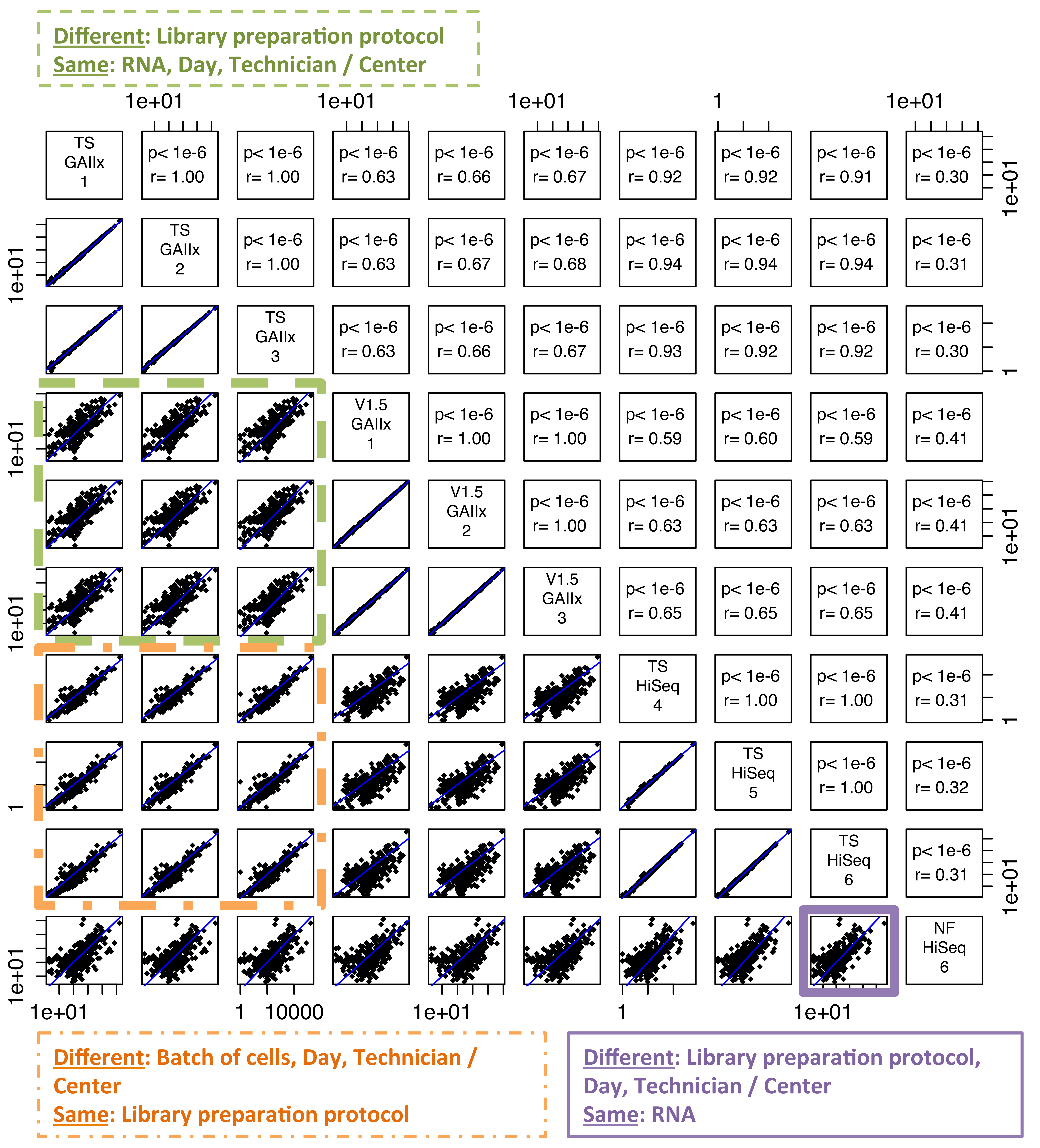

Supplement: Supplementary file 2 [file Image_1.TIFF]
